# The Galvanotactic Migration of Keratinocytes is Enhanced by Hypoxic Preconditioning

**DOI:** 10.1038/srep10289

**Published:** 2015-05-19

**Authors:** Xiaowei Guo, Xupin Jiang, Xi Ren, Huanbo Sun, Dongxia Zhang, Qiong Zhang, Jiaping Zhang, Yuesheng Huang

**Affiliations:** 1 Institute of Burn Research, State Key Laboratory of Trauma, Burns and Combined Injury, Southwest Hospital, The Third Military Medical University, Chongqing, China

## Abstract

The endogenous electric field (EF)-directed migration of keratinocytes (galvanotaxis) into wounds is an essential step in wound re-epithelialization. Hypoxia, which occurs immediately after injury, acts as an early stimulus to initiate the healing process; however, the mechanisms for this effect, remain elusive. We show here that the galvanotactic migration of keratinocytes was enhanced by hypoxia preconditioning as a result of the increased directionality rather than the increased motility of keratinocytes. This enhancement was both oxygen tension- and preconditioning time-dependent, with the maximum effects achieved using 2% O_2_ preconditioning for 6 hours. Hypoxic preconditioning (2% O_2_, 6 hours) decreased the threshold voltage of galvanotaxis to < 25 mV/mm, whereas this value was between 25 and 50 mV/mm in the normal culture control. In a scratch-wound monolayer assay in which the applied EF was in the default healing direction, hypoxic preconditioning accelerated healing by 1.38-fold compared with the control conditions. Scavenging of the induced ROS by N-acetylcysteine (NAC) abolished the enhanced galvanotaxis and the accelerated healing by hypoxic preconditioning. Our data demonstrate a novel and unsuspected role of hypoxia in supporting keratinocyte galvanotaxis. Enhancing the galvanotactic response of cells might therefore be a clinically attractive approach to induce improved wound healing.

To migrate effectively to heal a wound, keratinocytes must sense not only when to migrate but also the required direction. Endogenous electric fields (EFs) have been proposed as a directional cue guiding the migration of keratinocytes in wound healing^1^. Endogenous EFs are generated instantaneously after an injury due to the collapse of the trans-epithelial potentials, with the wound center being more negative than the surrounding tissue and thus acting as the cathode of the endogenous EF until wound re-epithelialization is complete^2^. Endogenous EFs measure between 42 and 100 mV/mm and are regulated spatially and temporally^1^,^2^. *In vitro*, many cell types respond to applied EFs at the strength equivalent to those measured *in vivo* by directional migration, a phenomenon termed galvanotaxis or electrotaxis^1^. Keratinocytes migrate toward the cathode in an applied EF *in vitro*, which is the same direction as migration occurs under an endogenous EF^2^,^3^,^4^,^5^,^6^,^7^. Significantly, recent studies found that EFs could override other cues in guiding cell migration during epithelial wound healing, indicating that the role for EFs in wound healing is far more important than previously thought^2^.

The wound microenvironment is, however, complex. Keratinocyte migration into wounds may therefore involve the integration of several relevant factors that co-exist in the wound. Although a number of membrane-bound and cytosolic proteins have been identified as being involved in the intracellular signaling that underpins keratinocyte galvanotaxis^2^,^3^,^4^,^5^,^6^, whether or how the mechanisms of galvanotaxis could be modulated by other factors in the wound microenvironment is mainly unknown. Hypoxia is a microenvironmental stress that occurs immediately after injury, likely due to the vascular disruption and increased oxygen consumption by cells at the wound’s edge^8^. The oxygen tension is approximately 0-10 mmHg (0-1.3% O_2_) in the wound center and shows an increased gradient toward the wound periphery, where it is approximately 60 mmHg (7.9% O_2_)^9^. As keratinocytes do not migrate immediately but a few hours post-wounding^10^, hypoxia may act as an initial stimulant for the migration of keratinocytes in a wound. In fact, hypoxia has been shown to increase the motility of both keratinocytes and fibroblasts *in vitro*^11^,^12^,^13^ and may play a beneficial role in wound re-epithelialization *in vivo*^10^,^14^. Wound re-epithelialization depends on the movement and, more importantly, the migratory direction of keratinocytes. However, the question of whether hypoxia is functional in terms of keratinocyte directionality when migrating over the wound site has not been addressed.

Numerous studies have described the EF-guided directional migration of keratinocytes *in vitro*^2^,^3^,^4^,^5^,^6^,^7^. Those studies, however, were all performed under normoxia, ignoring the wound-induced hypoxia that keratinocytes encounter prior to migration *in vivo*. Using a model of galvanotactic migration of keratinocytes, we here found the following: (1) the EF-guided directional migration of keratinocytes was enhanced by hypoxic preconditioning, with this effect resulting from the increased directionality rather than the motility of keratinocytes; (2) the enhanced keratinocyte galvanotaxis was both oxygen tension- and preconditioning time-dependent, with the maximum effects achieved using 2% O_2_ preconditioning for 6 hours; (3) hypoxic preconditioning decreased the threshold voltage required for keratinocyte galvanotaxis to < 25 mV/mm; (4) hypoxic preconditioning accelerated the healing process in a monolayer wound when the applied EF vector pointed in the default healing direction; and (5) ROS may be a link between hypoxic preconditioning and the enhanced galvanotaxis of keratinocytes.

## Results

### The characteristics of keratinocyte galvanotaxis

**Figure 1:**
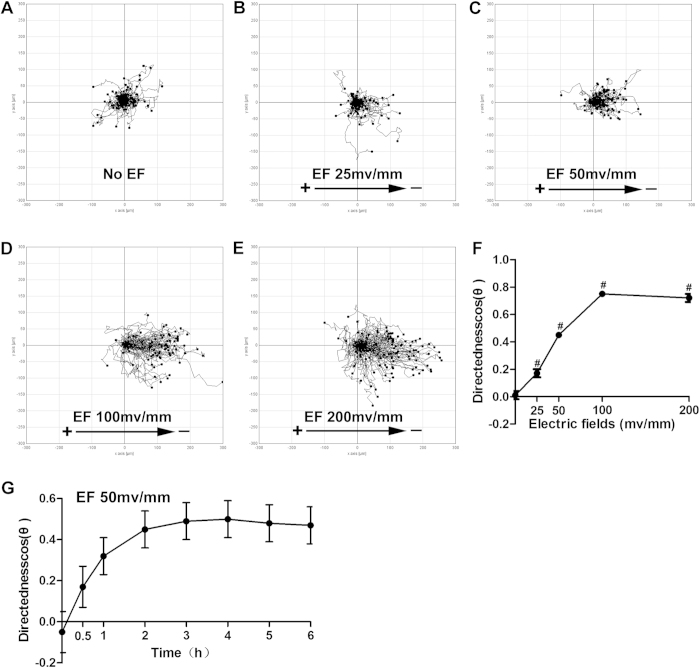
The characteristics of keratinocyte galvanotaxis. Keratinocytes were stimulated with or without direct current EFs (25, 50, 100 or 200 mV/mm) for 6 h (**G**) or 3 h (others). (**A**–**E**) The migration trajectories of keratinocytes guided by EF (**B**–**E**) or not (**A**), with the starting points positioned at the origin. (**F**) Quantitative analysis of directedness (cosθ) of keratinocyte migration. (**G**) The time course of keratinocyte directedness. The data are from at least 100 cells in 3 independent experiments and are shown as the mean ± SEM. #, p < 0.01 compared with the cells without EF stimulation.

To determine the optimal voltage and time of EF stimulation in our research, we first observed the EF-guided migration of keratinocytes under different voltages. The cathodal migration of keratinocytes was induced by EFs in a voltage-dependent manner (Fig. 1A–F), consistent with previous observations^4^,^7^,^15^. In an EF of 25 mV/mm, keratinocytes showed a migration directedness of 0.17 ± 0.03, a value that was lower than the threshold directedness (0.2) of cell directional migration, which was regarded as random migration (Fig. 1F). A sharp increase in the directedness of keratinocytes was observed at a field strength of 50 mV/mm, with a value of 0.45 ± 0.02 (Fig. 1F). The threshold voltage for keratinocyte galvanotaxis under our experimental conditions was therefore between 25 and 50 mV/mm. As a field strength of 50 mV/mm is comparable with the strength of endogenous wound EFs (42–100 mV/mm) as measured experimentally in animals and humans^2^, this strength was selected as the EF strength in our study to explore the role of hypoxia in keratinocyte galvanotaxis. The time course analysis revealed that the directionality of keratinocyte migration increased in a time-dependent manner, reaching a plateau 3 hours after exposure to an EF of 50 mV/mm (Fig. 1G). A stimulation time of 3 hours was therefore chosen as another experimental parameter in the following experiments.

### The galvanotactic migration of keratinocytes is promoted by hypoxic preconditioning

**Figure 2:**
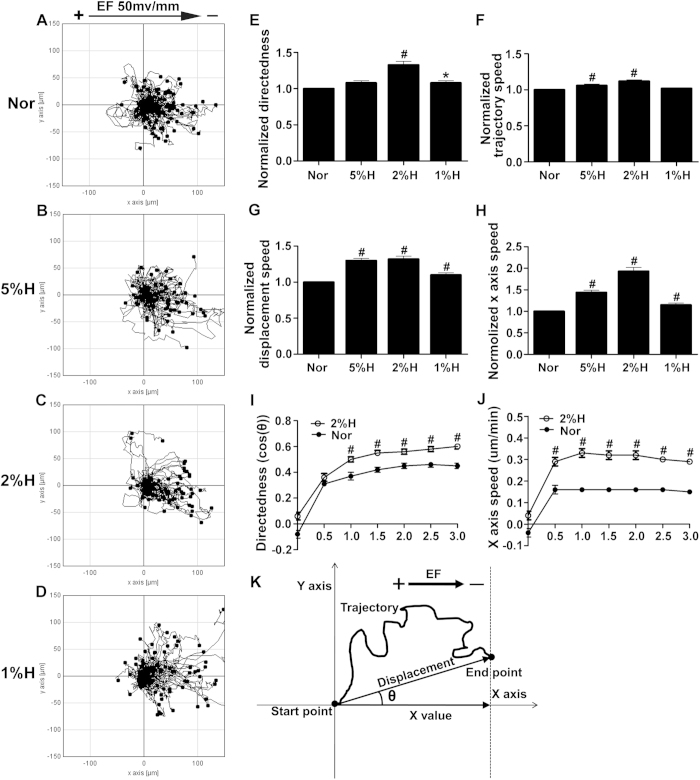
The galvanotactic migration of keratinocytes is promoted by hypoxic preconditioning. Keratinocytes were first pre-cultured in 1%, 2% or 5% O_2_ for 6 hours and then stimulated by an EF of 50 mV/mm for another 3 hours under normoxic conditions. (**A**–**D**) The migration trajectories of keratinocytes guided by EFs with hypoxic preconditioning (**B**–**D**) or not (**A**). (**E**–**H**) Analysis of the directedness (**E**), trajectory speed (**F**), displacement speed (**G**) and X-axis speed (**H**) of keratinocyte migration. (**I**–**J**) Time course of the directedness (**I**) and X-axis speed (**J**) of keratinocyte migration guided by EF under hypoxic preconditioning (2% O_2_, 6 hours) or normoxic culture conditions. (**K**) Illustration of the methods used for the quantification of cell migration. Also see the Materials and Methods section, “Quantitative analysis of cell migration” for details. The data are from at least 100 cells in 3 independent experiments and are shown as the mean ± SEM. *, p < 0.05; #, p < 0.01 compared with the cells without hypoxia pretreatment.

Oxygen tension changes during wound healing, ranging from 0–10 mmHg (0–1.3% O_2_) centrally to approximately 60 mmHg (7.9% O_2_) peripherally in skin wounds^9^. We examined the effects of hypoxic preconditioning at physiological levels on the EF-guided migration of keratinocytes. Keratinocytes were preconditioned in 1%, 2% or 5% O_2_ for 6 hours and then stimulated by an EF of 50 mV/mm for another 3 hours under normal culture conditions. Compared with the normoxia controls, hypoxic preconditioning of 1%, 2% or 5% O_2_ each promoted the EF-guided cathodal migration of keratinocytes to some extent, indicating that keratinocyte galvanotaxis could be modulated by hypoxic preconditioning (Fig. 2A–D, Supplemental Movie S1). Specifically, keratinocytes pretreated with 2% O_2_ showed a 33% increase in directedness, whereas 5% and 1% O_2_ caused 7.8% and 7.6% increases, respectively (Fig. 2E, K). Two percent O_2_ preconditioning caused a 13% increase in the trajectory speed, which represents the motility of keratinocytes, whereas 5% and 1% O_2_ preconditions resulted in 5.6% and 2.5% increases, respectively (Fig. 2F, K). Displacement speed, which represents the effective migration of keratinocytes, was increased by 32%, 29% or 10% after 2%, 5% or 1% O_2_ preconditioning, respectively (Fig. 2G, K). The X-axis speed, which is calculated as the cross-product between the displacement speed and directedness, represents an effective migration of keratinocytes along the EF vector *in vitro* or toward the wound center *in vivo*. This measure was increased by 93%, 45% or 17% following 2%, 5% or 1% O_2_ preconditioning, respectively (Fig. 2H, K). These results indicated that the positive effects of hypoxic preconditioning on EF-guided keratinocyte migration occurred in an oxygen tension-dependent manner, with the strongest effects achieved by 2% O_2_ preconditioning. The time course analysis revealed that the promoting effects of 2% O_2_ preconditioning on the directedness or X-axis speed of keratinocyte migration occurred as early as 0.5–1 hour and persisted up to 3 hours upon EF stimulation, further supporting a beneficial role for hypoxic preconditioning in keratinocyte galvanotaxis (Fig. 2I, J). Thus, hypoxia at physiological levels could not only promote the motility but also more effectively enhance the directional migration of keratinocytes. This finding might be of physiological significance given that keratinocytes initiate their migration under endogenous EFs in a hypoxic wound microenvironment.

### The threshold voltage for keratinocyte galvanotaxis is decreased by hypoxia preconditioning

**Figure 3:**
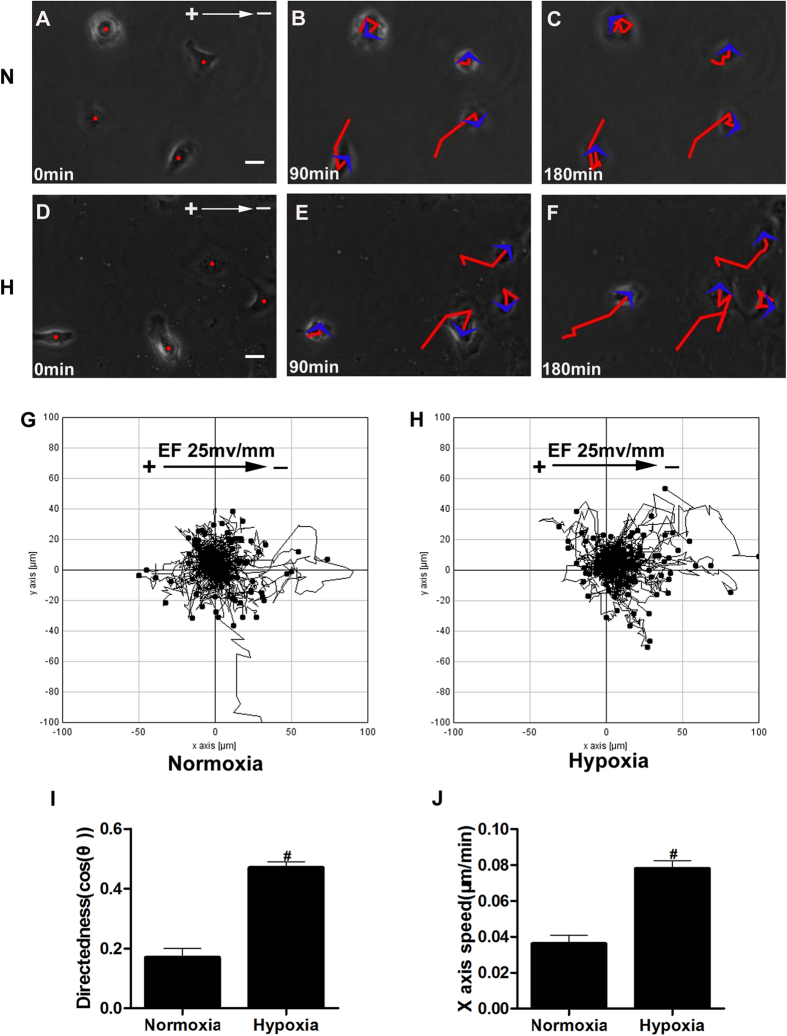
The threshold voltage for keratinocyte galvanotaxis is decreased by hypoxic preconditioning. Keratinocytes were pre-cultured in 2% O_2_ or under normoxic conditions for 6 hours and then exposed to an EF of 25 mV/mm for another 3 hours under normoxic conditions. (**A**–**F**) Time-lapse photographs of keratinocyte migration guided by EF with (**D**–**F**) or without (**A**–**C**) hypoxic preconditioning. The red lines with blue arrowheads represent the trajectories and direction of cell movement. (**G**–**H**) Migration trajectories of cells over 3 hours with (**H**) or without (**G**) hypoxic preconditioning. (**I**–**J**) Analysis of the directedness or X-axis speed of keratinocyte migration. The data are from at least 100 cells in 3 independent experiments and are shown as the mean ± SEM. #, p < 0.01 compared with the cells without hypoxia pretreatment. Bar = 25 μm.

We have determined that hypoxic preconditioning could enhance the migration of keratinocytes along the applied EFs. We next asked whether hypoxic preconditioning could increase the galvanotactic sensitivity of keratinocytes when stimulated with a low EF. The keratinocytes were pretreated with 2% O_2_ for 6 hours and then exposed to an EF of 25 mV/mm for another 3 hours. As mentioned above, the keratinocytes migrated randomly at an EF of 25 mV/mm under normal culture conditions (Fig. 1B, F). However, with hypoxic preconditioning, the keratinocytes exhibited a significant directional migration toward the cathode under the same EF (25 mV/mm, Fig. 3A–H, Supplemental Movie S2). Quantitative analysis showed that the directedness of 2% O_2_-preconditioned keratinocytes was as high as 0.47 ± 0.02, which is significantly higher than that observed in the controls (Fig. 3I) and comparable with that observed in keratinocytes exposed to an EF of 50 mV/mm under normal culture conditions (Fig. 1F). Similarly, the X-axis speed of keratinocytes was increased significantly by hypoxic preconditioning, with a value as high as nearly 2-fold that of the controls (Fig. 3J). These results suggested that hypoxic preconditioning increased the sensitivity of keratinocytes to a low applied EF and decreased the threshold voltage required for galvanotaxis to <25 mv/mm, whereas the required voltage was between 25 and 50 mV/mm in normal culture conditions (Fig. 1F). As the strength of endogenous EFs differs spatially *in vivo*, being much lower in the wound center and higher at the wound edge^16^, these results might therefore broaden our understanding of how the keratinocytes respond to a relatively low EF near the wound center but move over the wound effectively and site-directionally in a hypoxic wound microenvironment *in vivo*.

### Hypoxia-promoted keratinocyte galvanotaxis is preconditioning time-dependent

**Figure 4:**
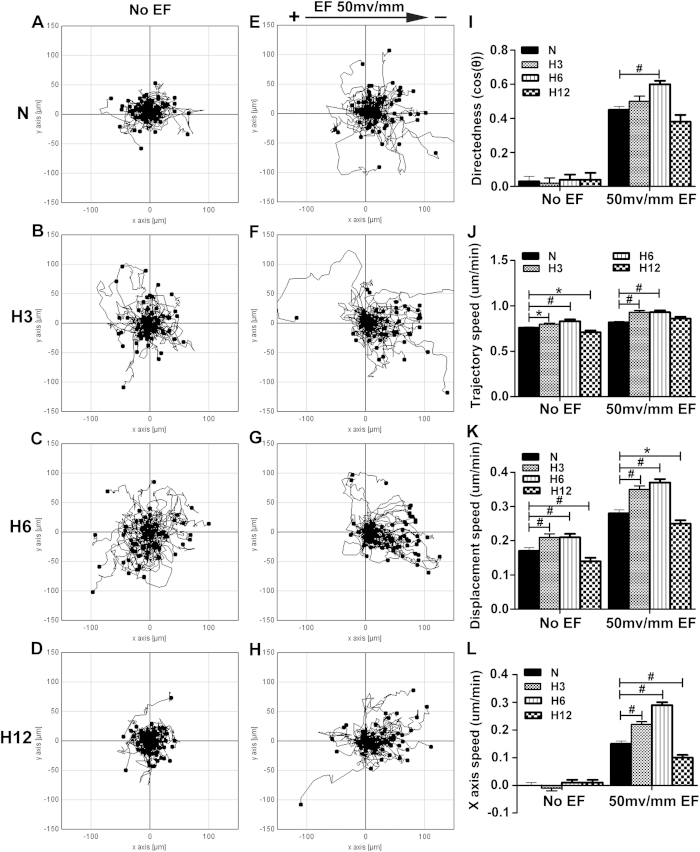
Hypoxia-promoted keratinocyte galvanotaxis is preconditioning-time dependent. The keratinocytes were exposed to 2% O_2_ for 3, 6 or 12 hours and then stimulated with an EF of 50 mV/mm for another 3 hours under normoxic conditions. (**A**–**H**) The migration trajectories of keratinocytes guided by EF (**E**–**H**) or not (**A**–**D**) under normoxic culture conditions (**A**, **E**) or 2% O_2_ preconditioning (**B**–**D**, **F**–**H**). (**I**–**L**) Analysis of the directedness, trajectory speed, displacement speed and X-axis speed of keratinocyte migration. The data are from at least 100 cells in 3 independent experiments and are shown as the mean ± SEM. *, p < 0.05; #, p < 0.01 compared with the cells without hypoxia pretreatment.

As long-term hypoxia is well known to impair wound repair, we determined the effects of various durations of hypoxic preconditioning on the EF-guided migration of keratinocytes. Keratinocytes were exposed to 2% O_2_ for 3, 6 or 12 hours and then stimulated with an EF of 50 mV/mm for another 3 hours under normal culture conditions. Compared with the normoxia control, the EF-guided directional migration of keratinocytes was promoted by 3 and 6 hours, but not 12 hours, of 2% O_2_ preconditioning (Fig. 4A–H, Supplemental Movie S3). Quantitative analysis showed that 3 and 6 hours of hypoxic preconditioning increased the directedness by 11% and 33%, respectively, whereas 12 hours of hypoxic preconditioning resulted in a 16% decrease in directedness (Fig. 4I). Hypoxic preconditioning also significantly increased the trajectory, displacement and X-axis speed in EF-stimulated keratinocytes, except at 12 hours (Fig. 4J–L), suggesting that hypoxia-enhanced keratinocyte galvanotaxis is preconditioning time-dependent. Notably, we found that the trajectory and displacement speed were moderately increased by 3 or 6 hours of hypoxia preconditioning for even non-EF-stimulated keratinocytes, although these measures were clearly attenuated by 12 hours of hypoxic preconditioning (Fig. 4J, K). This result indicated that a relatively long hypoxia treatment (i.e., 12 hours) may cause a general suppression of galvanotaxis; however, this effect was not unique to EF-induced migration. We then tested the activity of keratinocytes after various hypoxia treatments using CCK-8 and LDH assays. The CCK-8 assay was used to detect the growth of cells, and the LDH assay was used to estimate the viability of cells. As shown in Fig. S1A-B, keratinocyte activity was not changed by 3 or 6 hours but was significantly decreased following 12 hours of 2% O_2_ treatment. This finding provided a reasonable explanation for the observed dual role of hypoxic preconditioning in keratinocyte galvanotaxis in our study.

### Hypoxic preconditioning accelerates EF-guided keratinocyte migration in monolayer wounds

**Figure 5:**
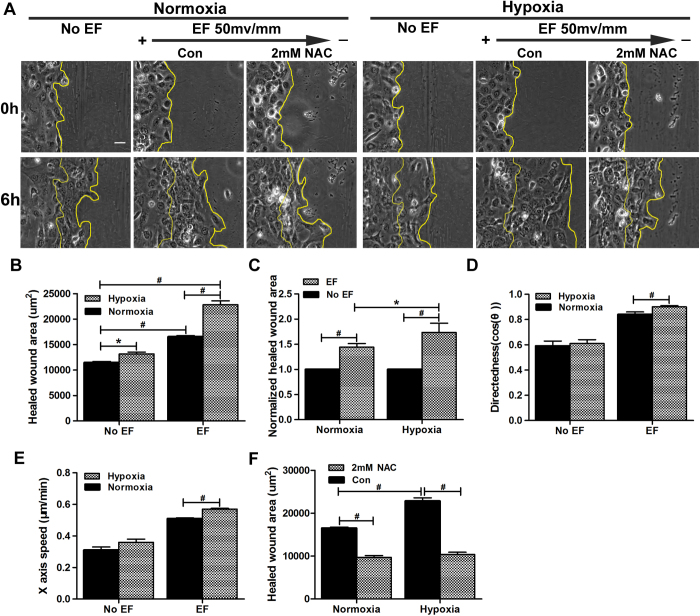
Hypoxic preconditioning accelerates EF-guided keratinocyte migration in monolayer wounds. Monolayer keratinocytes preconditioned by hypoxia (2% O_2_, 6 hours) or not were scratch-wounded with a P200 Gilson pipette tip and then exposed or not to an EF of 50 mV/mm for another 6 hours in the absence or presence of NAC. (**A**) Typical experimental images showing wound edges immediately following and 6 hours post-wounding. The wound edges are illustrated with a yellow line. (**B**) Analysis of healed wound areas. (**C**) Analysis of normalized healed wound areas. (**D**–**E**) Analysis of the directedness and X-axis speed of keratinocyte migration. (**F**) Analysis of the healed areas in the absence or presence of NAC. The data are from at least 3 independent experiments and are shown as the mean ± SEM. #, p < 0.01. Bar = 25 μm.

To test the physiological significance of hypoxia in keratinocyte galvanotaxis during wound healing, migration was monitored in a scratch-wounded monolayer under the stimulation of EF (50 mV/mm), with the vector pointing in the default healing direction, in the presence or absence of hypoxic preconditioning (2% O_2_, 6 hours). In the absence of an EF, hypoxic preconditioning accelerated the migration of keratinocytes into the wounds, as expected, with the healed areas increased by 1.15-fold compared with the normoxia control (Fig. 5A–B). This acceleration might be attributed to the increased motility of keratinocytes induced by hypoxia^12^,^13^. However, in the presence of an EF, hypoxic preconditioning accelerated the migration in healed areas by 1.38-fold compared with its corresponding control (Fig. 5A–B), indicating a synergistic effect between hypoxia and EF stimulation and that increased keratinocyte motility was not solely responsible for the accelerated wound healing. We performed another independent experiment in which the healed areas were normalized relative to a control monolayer that had no EF stimulation in the presence or absence of hypoxic preconditioning. Similarly, we found that the EF increased the healed areas by 1.74-fold in the hypoxia-preconditioned monolayer but only 1.44-fold in the normoxia-cultured monolayer (Fig. 5C), further confirming the synergistic role played by hypoxia and EF stimulation in contributing to the improved healing. Indeed, we found that the directionality and X-axis speed of EF-guided keratinocyte migration were all significantly increased in monolayers that were preconditioned by hypoxia (Fig. 5D–E). These results highlighted the physiological importance of hypoxia in the EF-guided migration of keratinocytes in wound healing (Supplemental Movie S4).

### ROS are linked to hypoxic preconditioning with enhanced keratinocyte galvanotaxis

**Figure 6:**
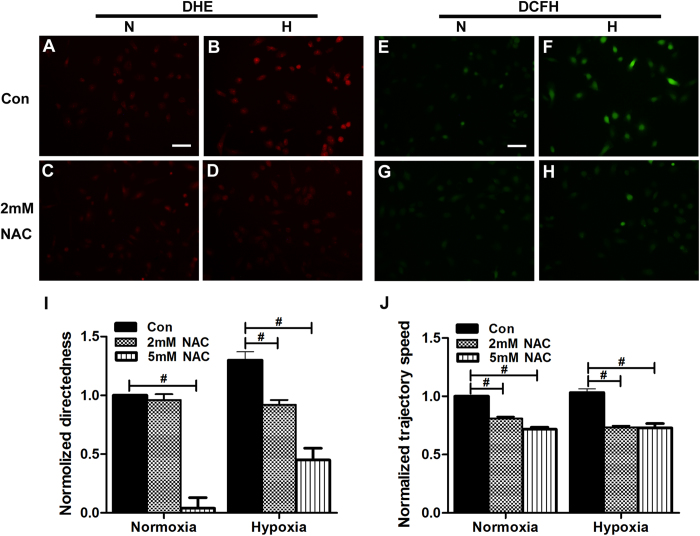
ROS are linked to hypoxic preconditioning with enhanced keratinocyte galvanotaxis. **(A**–**D**) Representative images showing the staining of intracellular superoxide by HE (red fluorescence) in keratinocytes in the absence or presence of NAC (2 mM) when preconditioned by hypoxia (2% O_2_, 6 hours) or not. (**E**–**H**) Representative images showing the staining of intracellular hydrogen peroxide by DCFH-DA (green fluorescence) in keratinocytes in the absence or presence of NAC (2 mM) when preconditioned by hypoxia (2% O_2_, 6 hours) or not. (**I**–**J**) Analysis of the normalized directedness (**I**) or trajectory speed (**J**) of keratinocyte migration guided by EF (50 mV/mm) in the absence or presence of NAC when the cells were preconditioned by hypoxia (2% O_2_, 6 hours) or not (normoxia). The data are from at least 100 cells in 3 independent experiments and are shown as the mean ± SEM. #, p < 0.01 compared with the cells treated with DMSO. Bar = 25 μm.

Reactive oxygen species (ROS), such as superoxide anion (O^2−^) and hydrogen peroxide (H_2_O_2_), are produced by the mitochondrial respiratory chain and released during hypoxia^17^. ROS have been considered important signals in the directional migration of cells^18^,^19^,^20^,^21^. We examined whether ROS are involved in hypoxic preconditioning-promoted keratinocyte galvanotaxis. To examine the intracellular ROS, keratinocytes were stained with the redox-sensitive dye DCFH-DA for the detection of hydrogen peroxide or with HE for the detection of superoxide. As shown in Fig. 6 A–B and E–F, the fluorescence signals of DCFH-DA and HE in keratinocytes were clearly increased by hypoxic preconditioning (2% O_2_, 6 hours), implying a robust production of ROS in keratinocytes. NAC (2 mM), a general pharmacological scavenger of ROS, reduced the fluorescence signal of both ROS to baseline levels (Fig. 6 C–D and G–H). In turn, the addition of NAC (2 mM) 30 minutes before EF stimulation abolished the promotion by hypoxic preconditioning of EF-guided keratinocyte directional migration, reducing the directionality to a level comparable with that observed in normal cultured keratinocytes (Fig. 6I, Supplemental Movie S5 A-D). This result suggested a tight association between the enhanced galvanotaxis and the induction of ROS by hypoxia. It should be noted that although the directionality of keratinocytes under normal culture conditions was not changed by NAC at 2 mM, it was sharply decreased by NAC at 5 mM (Fig. 6I). The cells thus lost their directionality and migrated randomly when exposed to the same EF (Supplemental Movie S5 E). This effect might be due to the over-scavenging of the basal ROS that are necessary for cell galvanotaxis (24). NAC at 5 mM also largely decreased the directedness of hypoxia-preconditioned keratinocytes (Fig. 6I, Supplemental Movie S5 F). Interestingly, although NAC at 2 or 5 mM resulted in a decrease in the motility of keratinocytes under EF stimulation in the presence or absence of hypoxic preconditioning, there was no significant difference between these treatments (Fig. 6J). These results imply a fundamental role for ROS in the electric cue-guided directional migration rather than the movement activity of keratinocytes. The addition of NAC (2 mM) also significantly reduced the healed areas in monolayers stimulated with EF in the presence or absence of hypoxic preconditioning (Fig. 5A, G; Supplemental Movie S4).

## Discussion

An essential role of endogenous wound EFs as guidance cues for the directional migration of keratinocytes has been well described^2^,^3^,^4^,^5^,^6^,^7^; the relevant experiments, however, were performed under normoxia, ignoring the hypoxic microenvironment that keratinocytes experience from time points even prior to their migration *in vivo*. We revealed for the first time that hypoxic preconditioning, depending on both the oxygen tension and the preconditioning time, promoted the galvanotactic migration and sensitivity of keratinocytes. Furthermore, hypoxic preconditioning accelerated the healing process in a scratch-wound monolayer assay when the applied EF vector pointed in the default healing direction. Scavenging of the induced ROS abolished the hypoxic preconditioning-induced enhancement of both keratinocyte galvanotaxis and monolayer wound healing. This result indicates a fundamental role of ROS in hypoxic preconditioning-enhanced galvanotaxis. Our study offers novel insights into the importance of hypoxia in wound healing.

Accumulating evidence indicates that hypoxia is an early stimulus for the initiation of wound re-epithelialization and tissue repair^10^,^11^. The question of how hypoxia contributes to the healing process remains unanswered, although hypoxia has been shown to be involved in the induction of cytokines, vascular permeability, and keratinocyte motility^10^. Using a model of keratinocyte galvanotaxis, we demonstrated that hypoxic preconditioning enhanced the galvanotactic migration of keratinocytes in an oxygen tension- and preconditioning time-dependent manner, with the maximum effects observed at 2% O_2_ preconditioning for 6 hours. Our results showed that a physiological small EF (50 mV/mm) followed by hypoxic preconditioning (2% O_2_, 6 hours) increased the directedness of keratinocytes by 33% (Fig. 2E). However, the trajectory speed of keratinocytes increased by 13% compared with keratinocytes treated only with the EF (Fig. 2F). This result indicates that hypoxia favors keratinocyte migration directionality more than motility when guided by EFs. As a consequence, the X-axis speed, which reflects the effective migration of keratinocytes along the applied EF vector *in vitro* or toward the wound center under endogenous EFs *in vivo*, was increased by 94% in hypoxia-preconditioned keratinocytes (Fig. 2H). This result might suggest a physiological importance of hypoxia in wound healing given that wound re-epithelialization depends on the directional migration of keratinocytes into the wound. To test this hypothesis directly, we designed an *in vitro* wound-scratch assay in keratinocyte monolayers, with the applied EF vector pointing in the default healing direction to mimic endogenous wound EFs. Using this model, we found that hypoxic preconditioning further accelerated the healing process compared with the use of the EF alone (Fig. 5A–C). This acceleration could not be explained simply by the promoted keratinocyte motility but was associated with the enhanced directionality and X-axis speed of keratinocyte migration after hypoxic preconditioning (Fig. 5D–E). In addition, we determined that hypoxic preconditioning could increase the galvanotactic sensitivity of keratinocytes. The threshold voltage of galvanotaxis was decreased to <25 mV/mm by hypoxic preconditioning, whereas this value was between 25 and 50 mV/mm in normal culture conditions (Fig. 1F and Fig. 3I). This finding further highlighted the importance of hypoxia in wound re-epithelialization, with the increased galvanotactic sensitivity potentially enabling the keratinocytes to effectively maintain a directional migration even if they are exposed to a relatively low EF somewhere in the wound. In this context, we propose a novel, previously unsuspected role for hypoxia in support of wound re-epithelialization by enhancing keratinocyte galvanotaxis.

Excess ROS are known to be harmful to cells, although ROS at low concentrations have been identified as ubiquitous intracellular messengers in signal transduction^22^. ROS have been shown to be important for the directional migration of neutrophils and hepatic pro-fibrogenic cells^18^,^19^. Recent studies also indicated a critical role for superoxide in the galvanotaxis of fibrosarcoma and glioma cells^20^,^21^. Consistent with these previous reports, we found that hypoxic preconditioning-enhanced keratinocyte galvanotaxis was associated with the induction of ROS. The addition of NAC, a general scavenger of ROS, at 2 mM suppressed the hypoxia-induced ROS to a basal level and abolished both the enhanced galvanotactic migration of keratinocytes and the accelerated healing of the monolayer wound (Fig. 6A–I and Fig. 5A, F). In addition, we found that although 2 mM NAC had little effect on the directedness of keratinocytes under normal culture conditions, 5 mM NAC resulted in a complete loss of galvanotaxis. The cells migrated randomly, with a moderate decrease in motility to a level similar to that observed following 2 mM NAC treatment (Fig. 6I–J). These results indicated an essential role for ROS in keratinocyte migration directionality rather than motility when guided by EF.

Although a beneficial role for hypoxia in the galvanotactic migration of keratinocytes has been described here, this effect was found to depend on both the oxygen tension and the preconditioning time. In our study, the most significant effects were observed following preconditioning in O_2_ for 6 hours. Although the relevant mechanisms must be investigated, the hypoxic response of cells has been reported to vary with the degree of hypoxia. For example, the production and deposition of collagen by fibroblasts have been proven to be proportional to the oxygen tension in the wound^10^. The regulation of hypoxia on human dermal fibroblast migration has also been shown to correlate with the oxygen content^11^. Considering that the oxygen tension in the *in vivo* wound microenvironment changes spatially and temporally^9^,^23^, the synergistic effects of wound hypoxia with endogenous EFs on keratinocyte migration would be more complex *in vivo* than that observed *in vitro*.

From a therapeutic standpoint, our finding may have clinical implications given that the manipulation of EFs has been shown to be a promising avenue to pursue improved wound healing. The exogenous application of EFs is a general approach that is expected to be beneficial for wound healing^24^,^25^,^26^,^27^. This approach, however, can be technically challenging because the application of exogenous EFs inevitably involves complications, such as electrode byproducts, heat generation, side effects on other cell types, and blood vessel permeability^28^. Another approach is to enhance endogenous EFs by selective manipulation of the ion transport required for EF generation; however, this approach has only been tested in the laboratory^2^. Based on our current study, we propose an attractive alternative that does not require EF modulation but that may enhance the galvanotactic response of cells and lead to improved wound healing.

In conclusion, we have determined, for the first time, that hypoxic preconditioning promotes keratinocyte galvanotaxis through the induction of ROS. These results provide novel insights into our understanding of the importance of hypoxia in wound re-epithelialization. Clinically, enhancing the galvanotactic response of cells might be an attractive approach for improved wound healing. In addition, wounded corneas and tumors are also simultaneously exposed to both hypoxic microenvironments and endogenous EFs. Whether our finding could be used in corneal wound healing and to prevent tumor metastasis is worth further investigation.

## Methods

### Ethics Statement

All of the animal procedures were approved by the Animal Experiment Ethics Committee of the Third Military Medical University and performed in accordance with the Guide for the Care and Use of Laboratory Animals published by the National Institutes of Health (NIH Pub. No. 85–23, revised 1996).

### Cell culture and reagents

Primary keratinocytes were obtained from skin of newborn BALB/c mice (postnatal day 1–3) as described previously^29^. Briefly, the keratinocytes was isolated from the skin by 0.25% trypsin/0.04% EDTA solution (Invitrogen, USA) at 4 °C overnight. The keratinocytes were then plated into dishes that were pre-coated with fibronectin (40 μg/mL) and cultured in serum-free medium (K-SFM medium) (Gibco, USA) supplemented with 100 U/ml penicillin (Invitrogen, USA), and 100 mg/ml streptomycin (Invitrogen, USA). The cells were incubated at 37 °C in 5% CO_2_ and 95% humidity for 24 hours and washed gently with warm phosphate-buffered saline (PBS) to discard non-adherent cells. The media were then refreshed. N-acetyl-l-cysteine (NAC) was obtained from Sigma-Aldrich (St. Louis, MO, USA). Dichlorofluorescein diacetate (DCFH-DA) and hydroethidine (HE) were purchased from Beyotime (cat. no. S0063, Haimen, China).

### EF stimulation and the imaging of cell migration and monolayer wound healing

For the cell migration assays, the cells were seeded at a density of 0.5 × 10^4^/cm^2^. A previously developed chamber^2^ was used to stimulate cells with electric fields. The EFs were used to stimulate cells through two silver electrodes in beakers containing Steinberg’s solution (60 mM NaCl, 0.7 mM KCl, 0.8 mM MgSO_4_, 0.3 mM CaNO_3_·4H_2_O, and 1.4 mM Tris base, pH 7.4) that were connected to pools of excess culture medium on each side of the galvanotaxis chamber by two agar bridges (2% agar in Steinberg’s solution). The media were supplemented with 20% fetal bovine serum in the EF stimulation experiments. The strength of the EFs was measured directly at the beginning and end of the experiment. The keratinocytes were exposed to EFs with strengths of 0, 25, 50, 100 and 200 V/mm for 3 or 6 hours. The time-lapse imaging was performed with a Zeiss imaging system (Carl Zeiss Meditec, Jena, Germany) that was equipped with a CO_2_- and temperature-controlled chamber, and the images were acquired every 3 minutes. Time-lapse images were analyzed using NIH ImageJ software.

### Hypoxia pretreatment

The hypoxic conditions were created using a Forma Series II Water Jacket CO_2_ incubator (model: 3131; Thermo Scientific), which allows precise oxygen and temperature regulation. The hypoxic conditions were generated at 37 °C in 5% CO_2_ and the designated oxygen content, balanced with N_2_. All of the media used in the hypoxia experiments were preincubated overnight in the chambers with the designated oxygen content.

### Monolayer wound healing assays

Monolayer wound healing assay was performed as described previously^30^. For the monolayer wound healing assays, the cells were seeded at a density of 3 × 10^4^ /cm^2^ and cultured until confluent. Upon reaching full confluence, the cells were damaged by scraping off the monolayer, with the wounding being perpendicular to the applied EF vector using a P200 Gilson pipette tip. The medium and cell debris were then washed out, and fresh medium was added. The EF was then applied, and keratinocyte movement and wound healing were imaged.

### Detection of intracellular levels of ROS

The intracellular ROS levels were detected with a fluorescence microscope using DCFH-DA and HE. DCFH-DA was used to monitor intracellular-produced hydrogen peroxide (H_2_O_2_)^31^. HE is a good monitor of intracellular superoxide (O_2_^•−^) because it can be oxidized rapidly to the fluorescent molecule ethidium bromide by O_2_^•−^.^32^ In our study, DCFH-DA (5 μM) and HE (5 μM) were added to the cells after culture under hypoxic or normoxic conditions. After a 20-minute incubation at 37 °C, the cells were quickly washed twice with PBS to remove the extra dye. The fluorescence of the cells was observed and imaged under a fluorescence microscope with an excitation wavelength of 488 nm and an emission wavelength of 530 nm for DCFH-DA. For HE, these wavelengths were 510 nm and 600 nm, respectively.

### Quantitative analysis of cell migration

Cell migration was analyzed as previously reported^15^ and is illustrated in Fig. 2K. We used NIH ImageJ software to quantify the galvanotaxis and motility of cells. We traced the position of cell nuclei at frame intervals of 6 minutes and positioned the starting points at the origin. The directedness of cell migration was expressed as a function of cosθ, where θ is the angle between the EF vector and a straight line from the origin to the end position of a cell. The cosθ was calculated using the x value of the end position divided by the displacement of the cell migration.Values close to 0 represent random cell movement, and those close to 1 represent cells moving toward the cathode. Values close to −1 represent cells moving toward the anode. The values of cosθ range from −1 to +1 and objectively quantify the direction of cell migration. The X -axis speed is the x value of the end position divided by time, representing the migration speed along the EF vector. The directedness multiplied by the displacement speed reflects the directional migration speed of cells. Displacement speed is the straight-line distance between the start and end positions of a cell divided by time. The trajectory speed is the total length of the trajectory divided by time, reflecting cell motility.

### Statistical analysis

The data are expressed as the mean ± standard error of mean (SEM). Two-tailed Student’s t-tests were performed to determine significant differences in cell migration between groups. P < 0.05 was considered statistically significant.

## Author Contributions

Y.H. and J.Z. supervised the study. J.Z., Y.H. and X.G. developed the initial concept. X.G., X.J., J.Z. and Y.H. designed experiments. X.G., X.J., X.R., H.S., D.Z. and Q.Z. performed the experiments and analyzed the data. X.G., J.Z. and Y.H. co-wrote the manuscript. All of the authors discussed the results and commented on the manuscript.

## Additional Information

**How to cite this article**: Guo, X. *et al*. The Galvanotactic Migration of Keratinocytes is Enhanced by Hypoxic Preconditioning. *Sci. Rep.* **5**, 10289; doi: 10.1038/srep10289 (2015).

## Supplementary Material

Supplementary Information

Supplementary Movie S1

Supplementary Movie S2

Supplementary Movie S3

Supplementary Movie S4

Supplementary Movie S5
