# Supplementary material for: The Galvanotactic Migration of Keratinocytes is Enhanced by Hypoxic Preconditioning
Source: Sci Rep. 2015 May 19;5:10289. doi: 10.1038/srep10289 (PMC4437307; doi:10.1038/srep10289)
Supplement: Supplementary Information [file srep10289-s1.doc]

**The Galvanotactic Migration of Keratinocytes is Enhanced by Hypoxic Preconditioning**

Xiaowei Guo, Xupin Jiang, Xi Ren, Huanbo Sun, Dongxia Zhang, Qiong Zhang, Jiaping Zhang & Yuesheng Huang

Institute of Burn Research, State Key Laboratory of Trauma, Burns and Combined Injury, Southwest Hospital, The Third Military Medical University, Chongqing, China.

Correspondence and requests for materials should be addressed to Y.H. (yshuang.tmmu@gmail.com) or J.Z. (japzhang@aliyun.com).

**Supplementary Information**

**Supplementary Methods Information**

**Cell Counting Kit-8 assay.** The keratinocytes were seeded at a density of 8×10^3^/well in 96-well plates and left to attach overnight. Non-adherent cells were then discarded, and the media were refreshed. Cell activity following exposure to hypoxia and normoxia conditions was measured by CCK-8 (Dojindo Molecular Technologies, Kumamoto, Japan) according to the manufacturer's instructions. A CCK-8 solution (10 µL) was added to each well of the plate, and the plate was then incubated for 2 hours at 37°C. Lastly, the amount of formazan dye was measured at 450 nm using a microplate reader (Thermo, USA). All of the experiments were performed in eight replicates and repeated three times.

**Lactate dehydrogenase (LDH) cytotoxicity assay.** Cell activity was assessed using the LDH cytotoxicity assay. The keratinocytes were cultured in 96-well plates (8×10^3^/well). The culture medium was replaced with assay medium (1% fetal bovine serum) prior to hypoxia pretreatment. After pretreatment, the LDH cytotoxicity assay was performed using the LDH Cytotoxicity Assay kit (cat. no. C0016, Beyotime, Haimen, China), following the manufacturer’s instructions. Briefly, after the cell culture plate was centrifuged in a microplate-centrifuge at 400×g for 5 minutes, 120 µL of the supernatant from each well was transferred into a new 96-well plate, and 60 µL of LDH substrate was added to each well. After a 30-minute incubation in the dark at room temperature, the absorbance was read at 490 nm using a microplate reader (Thermo, USA). All of the experiments were performed in eight replicates and repeated three times.

**Supplementary Figure**

**
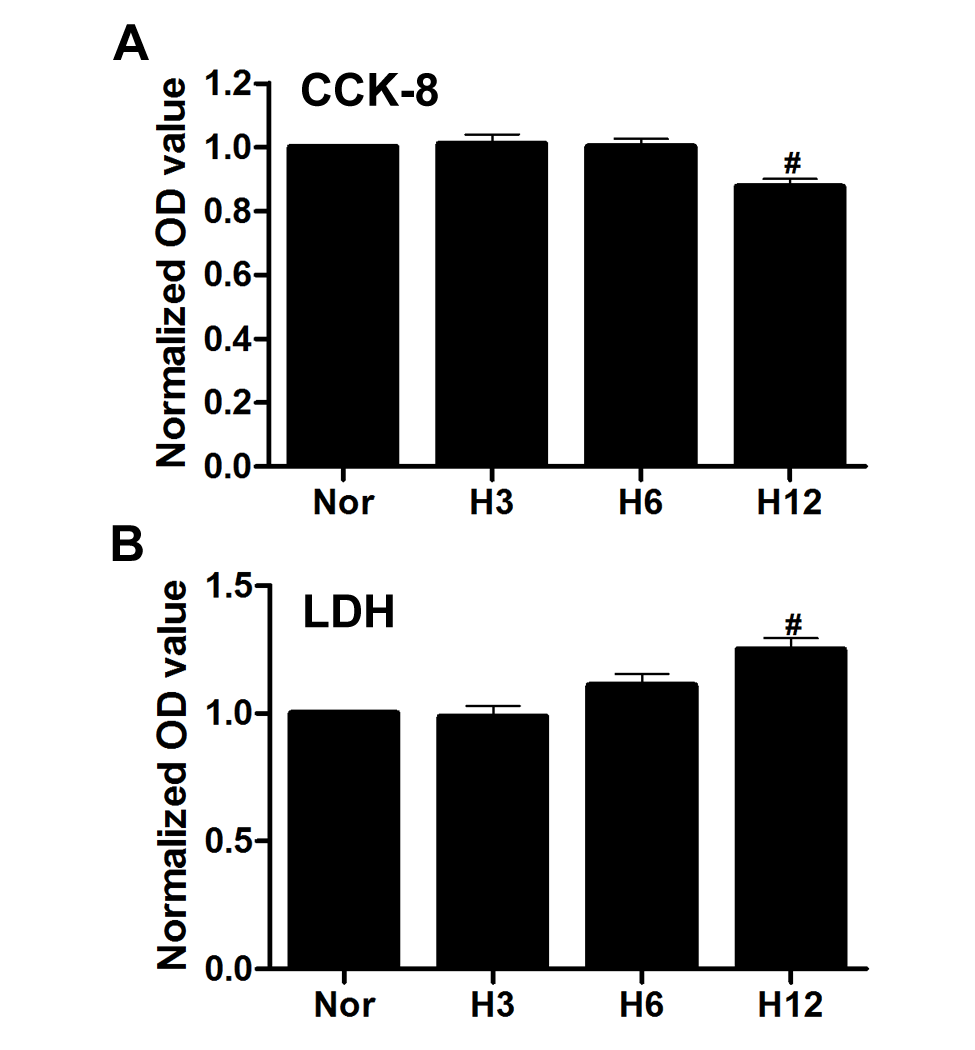
**

**Figure S1 Activity of keratinocytes after hypoxic preconditioning.** (A, B) Keratinocyte activity was assessed by the CCK-8 (A) and LDH cytotoxicity assays (B) after pre-culture in 2% O_2_ for 3, 6 or 12 hours. The data are from 3 independent experiments and are shown as the mean ± SEM. #, p<0.01 compared with the cells without hypoxia pretreatment.

**Supplementary Movie Legends**

**Supplemental Movie S1 Galvanotactic migration of keratinocytes is promoted by hypoxic preconditioning.** Keratinocytes were first pre-cultured in 1%, 2% or 5% O_2_ for 6 hours and then stimulated by an EF of 50 mV/mm for another 3 hours under normoxic conditions. The lines with yellow arrowheads represent the trajectories and direction of cell movement.

**Supplemental Movie S2 The threshold voltage for keratinocyte galvanotaxis is decreased by hypoxic preconditioning.** Keratinocytes were pre-cultured or not in 2% O_2_ for 6 hours and then exposed to an EF of 25 mV/mm for another 3 hours under normoxic conditions. The lines with yellow arrowheads represent the trajectories and direction of cell movement.

**Supplemental Movie S3 Hypoxia-promoted keratinocyte galvanotaxis reaches the maximum effects using 2% O_2_ preconditioning for 6 hours.** The keratinocytes were exposed to 2% O_2_ for 6 hours and then stimulated by an EF of 50 mV/mm for another 3 hours under normoxic conditions. The lines with yellow arrowheads represent the trajectories and direction of cell movement.

**Supplemental Movie S4 Hypoxic preconditioning accelerates EF-guided keratinocyte migration in a monolayer wound assay.** Monolayer keratinocytes preconditioned by hypoxia (2% O_2_, 6 hours) or not were scratch-wounded and then exposed to an EF of 50 mV/mm or not for another 6 hours after NAC pretreatment (2 mM) or not. The wound edges are illustrated with thin yellow lines at time point 0 and with thick yellow lines at time point 6 hours.

**Supplemental Movie S5 ROS is linked to hypoxic preconditioning with enhanced keratinocyte galvanotaxis.** Keratinocytes preconditioned by hypoxia (2% O_2_, 6 hours) or not were treated with 2 or 5 mM NAC or not and then stimulated with an EF of 50 mV/mm for another 3 hours under normoxic conditions. The lines with yellow arrowheads represent the trajectories and direction of cell movement.
